# Dynamic Regulation of Extracellular Superoxide Production by the Coccolithophore *Emiliania huxleyi* (CCMP 374)

**DOI:** 10.3389/fmicb.2019.01546

**Published:** 2019-07-12

**Authors:** Sydney Plummer, Alexander E. Taylor, Elizabeth L. Harvey, Colleen M. Hansel, Julia M. Diaz

**Affiliations:** ^1^ Department of Marine Sciences, Skidaway Institute of Oceanography, University of Georgia, Savannah, GA, United States; ^2^ Department of Chemistry, University of Vermont, Burlington, VT, United States; ^3^ Department of Marine Chemistry and Geochemistry, Woods Hole Oceanographic Institution, Woods Hole, MA, United States

**Keywords:** reactive oxygen species, superoxide, *Emiliania huxleyi*, photophysiology, oxidative stress, redox homeostasis, biogeochemical cycling

## Abstract

In marine waters, ubiquitous reactive oxygen species (ROS) drive biogeochemical cycling of metals and carbon. Marine phytoplankton produce the ROS superoxide (O_2_^−^) extracellularly and can be a dominant source of O_2_^−^ in natural aquatic systems. However, the cellular regulation, biological functioning, and broader ecological impacts of extracellular O_2_^−^ production by marine phytoplankton remain mysterious. Here, we explored the regulation and potential roles of extracellular O_2_^−^ production by a noncalcifying strain of the cosmopolitan coccolithophorid *Emiliania huxleyi*, a key species of marine phytoplankton that has not been examined for extracellular O_2_^−^ production previously. Cell-normalized extracellular O_2_^−^ production was the highest under presumably low-stress conditions during active proliferation and inversely related to cell density during exponential growth phase. Removal of extracellular O_2_^−^ through addition of the O_2_^−^ scavenger superoxide dismutase (SOD), however, increased growth rates, growth yields, cell biovolume, and photosynthetic efficiency (*F_v_/F_m_*) indicating an overall physiological improvement. Thus, the presence of extracellular O_2_^−^ does not directly stimulate *E. huxleyi* proliferation, as previously suggested for other phytoplankton, bacteria, fungi, and protists. Extracellular O_2_^−^ production decreased in the dark, suggesting a connection with photosynthetic processes. Taken together, the tight regulation of this stress independent production of extracellular O_2_^−^ by *E. huxleyi* suggests that it could be involved in fundamental photophysiological processes.

## Introduction

Ubiquitous reactive oxygen species (ROS) within marine waters help drive global biogeochemical cycling. ROS include intermediates in the reduction of molecular oxygen (O_2_) to water (H_2_O), which consist of superoxide ($O_{2}^{-}$), hydrogen peroxide (H_2_O_2_), and hydroxyl radical (OH•). These ROS shape the transformation of metal nutrients including iron (Rose, 2012) and manganese (Wuttig et al., 2013), as well as carbon (Heller and Croot, 2010b) due to their ability to act as both oxidants and reductants. Within aquatic environments, ROS are produced through both abiotic (e.g., *via* photodegradation of organic matter) and biotic means (Zinser, 2018). Biotic mechanisms include active extracellular production by marine microorganisms, which can be a dominant source of O_2_^−^ in natural waters (Rose et al., 2008; Hansard et al., 2010).

While ROS are formed intracellularly within all aerobic organisms as metabolic by-products, a plethora of marine microorganisms actively produce ROS extracellularly as well. Although intracellular O_2_^−^ can be released into the marine environment upon cell lysis, these rates cannot account for the steady-state concentrations that have been measured in natural waters (Rose, 2012). Furthermore, within cells, O_2_^−^ exists in equilibrium with its conjugate acid, the hydroperoxyl radical (HOO•); however, with a pK_a_ of 4.8, the O_2_^−^ anion is the dominant form at physiological pH (Bielski et al., 1985). Unlike H_2_O_2_, O_2_^−^ cannot readily diffuse across membranes due to its negative charge, short lifetime (~10^−5^ s), and limited diffusive distance (~10^−7^ m) (Lesser, 2006; Brown and Griendling, 2009; Diaz and Plummer, 2018). Thus, the majority of microbially derived O_2_^−^ within the extracellular environment must be created on or near the cell surface via active extracellular O_2_^−^ production mechanisms (Diaz and Plummer, 2018).

The ability to produce extracellular O_2_^−^ has been documented among heterotrophic bacteria (Diaz et al., 2013) and phytoplankton, including cyanobacteria (Rose et al., 2005, 2008; Godrant et al., 2009; Hansel et al., 2016), diatoms (Kustka et al., 2005; Hansel et al., 2016; Schneider et al., 2016), dinoflagellates (Saragosti et al., 2010; Zhang et al., 2016a), nontoxic microalgae (Marshall et al., 2005a), and harmful microalgae (Oda et al., 1997; Marshall et al., 2005a,b; Portune et al., 2010; Diaz and Plummer, 2018). Despite advancements in identifying the extensive presence and environmental relevance of extracellular O_2_^−^ production by marine microflora, the mechanisms of extracellular O_2_^−^ production and its biological roles are not well understood. The freshwater chlorophyte *Chlamydomonas reinhardtii* (Anderson et al., 2015) and marine raphidophytes *Chattonella marina* and *Chattonella ovata* (Kim et al., 2000, 2007) are either confirmed or thought to produce extracellular O_2_^−^ via cell membrane associated enzymes known as NADPH oxidases (Nox). These enzymes transfer electrons from cytosolic NADPH pools across cell membranes to reduce O_2_ in the surrounding aqueous environment, thus creating extracellular O_2_^−^. The presence of these enzymes has been implicated in diatoms and dinoflagellates as well (Kim et al., 2000; Kustka et al., 2005; Hervé et al., 2006; Saragosti et al., 2010). Extracellular O_2_^−^ production is light dependent in several phytoplankton taxa (Kim et al., 1999; Marshall et al., 2002; Milne et al., 2009; Saragosti et al., 2010; Hansel et al., 2016; Schneider et al., 2016), which has led to speculation that photosynthesis may serve an indirect role in extracellular O_2_^−^ production by supplying NADPH to cell surface-associated NADPH-oxidizing enzymes such as Nox (Marshall et al., 2002; Saragosti et al., 2010; Schneider et al., 2016).

Proposed biological roles of phytoplankton-derived extracellular O_2_^−^ are diverse (Diaz and Plummer, 2018). For instance, extracellular O_2_^−^ production has been implicated in harmful algal bloom toxicity (Tanaka et al., 1992; Yang et al., 1995; Kim et al., 1999; Marshall et al., 2003; Kim and Oda, 2010; Dorantes-Aranda et al., 2013, 2015; Mardones et al., 2015), metal nutrient acquisition (Rose et al., 2005; Garg et al., 2007; Liu et al., 2007; Rose, 2012; Roe and Barbeau, 2014), allelopathy (Oda et al., 1992, 1997; Marshall et al., 2005b), and defense against grazing (Martel, 2009; Flores et al., 2012). Model phytoplankton species generate abundant extracellular O_2_^−^ even under ideal growth conditions in the absence of any obvious stressors (Kustka et al., 2005; Rose et al., 2005; Marshall et al., 2005a,b; Godrant et al., 2009; Portune et al., 2010; Diaz et al., 2013; Hansel et al., 2016; Schneider et al., 2016), suggesting an association with basal functioning. For example, extracellular O_2_^−^ regulates growth and morphology in the prolific ROS producer, *C. marina* (Oda et al., 1995), as well as growth in bacteria (Saran, 2003; Hansel et al., 2019) and differentiation in microbial eukaryotes (Aguirre et al., 2005). In these microorganisms, extracellular O_2_^−^ production rates are the highest during active growth and at low cell densities consistent with beneficial cell signaling and autocrine growth regulation, as also seen in plants (Mittler et al., 2011) and animals (Brown and Griendling, 2009; Aguirre and Lambeth, 2010). The accumulation of studies showing that extracellular $O_{2}^{-}$ production by diverse phytoplankton is similarly dependent on cell density (Marshall et al., 2005a; Hansel et al., 2016; Diaz et al., 2018) and growth phase (Oda et al., 1995; Kim et al., 1999; Portune et al., 2010) has spurred speculation that extracellular O_2_^−^ production may be involved in phytoplankton cell signaling and/or growth regulation in species other than *C. marina*, although this possibility remains largely untested (Hansel et al., 2016; Diaz and Plummer, 2018).

Clarifying the cellular regulation and biological function of active extracellular O_2_^−^ production by phytoplankton is critical to understand the effects of ROS on ocean redox balance, biogeochemical cycling, and ecological interactions in marine waters. Among phytoplankton, coccolithophores are one of the most prevalent groups in the global ocean. Further, the original report of extracellular H_2_O_2_ production by the coccolithophorid species *Pleurochrysis carterae* pioneered the recognition of microorganisms as significant sources of ROS in aquatic systems (Palenik et al., 1987). Despite this discovery, however, coccolithophorids are under explored in terms of extracellular ROS production. Therefore, this study was conducted to investigate the dynamics, cellular regulation, and biological function of extracellular O_2_^−^ production by a noncalcifying strain of *Emiliania huxleyi* (CCMP 374), the most prevalent coccolithophore species in modern oceans (Westbroek et al., 1989; Brown and Yoder, 1994).

## Materials and Methods

### Cultivation of *E. huxleyi*, Growth Tracking, and Cell Counts

Axenic cultures of *E. huxleyi* CCMP 374 were obtained from the National Center for Marine Algae and Microbiota (NCMA) at Bigelow Laboratory for Ocean Sciences (East Boothbay, ME). Cultures of *E. huxleyi* were inoculated into f/2 growth media prepared without the addition of silicic acid (Guillard and Ryther, 1962) using 0.2 μm filtered natural seawater collected from the South Atlantic Bight. Media were prepared and autoclaved (121°C, 20 min) at least 1 day prior to inoculating cultures. Cultures were begun with exponential phase inocula, unless otherwise stated. Cultures were either grown in borosilicate culture tubes with caps or Erlenmeyer flasks of various sizes with aluminum foil or an acid washed plastic beaker covering the mouth of the flask at 18°C under cool, white light (~130 μmol photons m^−2^ s^−1^, 14:10 light dark cycle). Growth was monitored by observing *in vivo* chlorophyll fluorescence using an AquaFluor^®^ handheld fluorometer (Turner Designs, San Jose, CA) or a 10-AU™ fluorometer (Turner Designs, San Jose, CA). *In vivo* fluorescence values were normalized to measurements taken on day 0. Exponential growth phase was defined as the log-linear portion of the *in vivo* fluorescence data versus time (*R*^2^ ≥ 0.98 in all cultures). Stationary phase was determined to be the time between the end of the log linear portion of the growth curve and until the end of the growth curve. Specific growth rates during exponential growth phase were found by calculating the slope of the regression of the natural log-normalized *in vivo* fluorescence versus time. Culture pH was monitored using an Accumet AB 15/15+ pH meter (Thermo Fisher Scientific, Waltham, MA) in cultures grown from stationary phase inocula in 25 mm borosilicate tubes (Thermo Fisher Scientific, 14-961-34). *E. huxleyi* cell abundances (cells ml^−1^) were obtained using a Guava^®^ easyCyte flow cytometer (Millipore Sigma, Merck KGaA, Dermstadt, Germany) and analyzed with Guava InCyte™ 3.1 software. Flow cytometry samples were preserved with a final concentration of 0.5% glutaraldehyde, as well as 1% peptone to prevent cell adsorption to sample tubes. Flow cytometry samples were stored at −80°C prior to processing. To process samples, 50–200 μl of each sample was pipetted into 96-well plates, diluted with filtered seawater as needed, and run at a low flow rate (0.24 μl s^−1^) for 3 min. For analysis, concentrations of healthy cell populations (cells ml^−1^) were determined based on gates of red fluorescence and forward scatter signals from previously run samples of exponentially growing cultures.

In two separate experiments, extracellular O_2_^−^ was removed from *E. huxleyi* cultures by adding superoxide dismutase (SOD, Millipore Sigma 574,594-50KU), an enzyme that specifically degrades O_2_^−^. To begin this experiment, 7.5 ml of media were inoculated with 300 μl stationary phase culture to give an initial concentration of ~2.3 × 10^5^ cells ml^−1^ and grown under the conditions above in 13 mm borosilicate glass tubes (Thermo Fisher Scientific, 14-962-26D). Treatments included three different concentrations of SOD added at three different volumes and a deionized water (DI) control. A 10 kU ml^−1^ stock of SOD was prepared using DI. Then, treatments were performed on triplicate cultures, where each tube received daily additions of 20.25 μl, 37.5 μl, or 75 μl of the 10 kU ml^−1^ SOD stock, or 75 μl DI to give final concentrations of 27 U ml^−1^ SOD, 50 U ml^−1^ SOD, 100 U ml^−1^ SOD, or 0 U ml^−1^ SOD, respectively. A subsequent control experiment was performed with diafiltered SOD to ensure that SOD was responsible for potential changes observed in the cultures. For this control experiment, 7.5 ml of media were inoculated with 300 μl stationary phase culture to give an initial concentration of ~1.8 × 10^5^ cells ml^−1^ and grown under the conditions above in 13 mm borosilicate glass tubes. To create the dialyzed SOD, a 10 kU ml^−1^ SOD stock prepared with DI was diafiltered by passing the SOD solution through an Amicon ultrafiltration device (10 kDa molecular weight cut-off, Millipore) at 3200 ×*g* for 20 min at 4°C, thereby removing the enzyme from the solution. Treatments were performed on triplicate cultures, where each tube received daily additions of 75 μl dialyzed SOD, 75 μl DI, and 75 μl SOD (100 U ml^−1^ SOD final concentration).

### Cell Imaging and Analysis

Individual *E. huxleyi* cells were imaged using a FlowCam^®^ (Fluid Imaging Inc., Scarborough, ME), a continuous flow-through microscope fitted with a color and monochromatic camera. To calibrate the FlowCam^®^ for imaging, dilutions of *E. huxleyi* cultures from 0 to 95% using 0.2 μm filtered seawater were analyzed in varying context settings. Final settings (Supplementary Table S1) were chosen based on their ability to differentiate cell shape and color and provide useable images for morphological and volume assessments. Culture samples of 100 μl were diluted with 500 μl of 0.2 μm filtered seawater prior to analysis on the FlowCam^®^. Images were analyzed with VisualSpreadsheet^®^ (Fluid Imaging Inc., Scarborough, ME). The spherical biovolume was determined using three separate measurements of cell diameter. The FlowCam^®^ software calculates three different diameters for each cell image [area based diameter (ABD), equivalent spherical diameter (ESD), filled or full diameter (FD)], thus giving three alternative biovolume assessments for every imaged cell. Further analysis and filtering of FlowCam^®^ images as well as details on diameter calculations are described in the Supplementary Material.

### Photophysiology

Photophysiological health of *E. huxleyi* was monitored using the Satlantic fluorescence induction and relaxation (FIRe) fluorometer system (Sea-bird Scientific, Halifax, NS Canada). Prior to analysis, samples were allowed to dark adapt for approximately 30 min and were then diluted using 0.2 μm filtered seawater as necessary to avoid detector saturation. Samples were illuminated with a single turnover flash of blue excitation for a duration of 80 μs. The measured fluorescence response was used to calculate the maximum efficiency of photosystem II (PSII), or *F_v_/F_m_*, using the equation:

$$F_{v}/F_{m}=\;\;\;\frac{\left(F_{m}-F_{o}\right)}{F_{m}}$$

where *F*_*m*_ is the maximum fluorescence yield, and *F*_*o*_ is the minimum fluorescence yield. For each sample, 20 acquisitions were obtained.

### Extracellular $O_{2}^{\;-}$ Production

Net extracellular O_2_^−^ production by *E. huxleyi* was measured using the flow-through FeLume (II) analytical system (Waterville Analytical, Waterville, ME) by detecting chemiluminescence emitted through the reaction of O_2_^−^ and the specific probe methyl *Cypridina* luciferin analog (MCLA), as previously described (Diaz et al., 2013; Schneider et al., 2016). This method allows for manipulation of experimental settings (e.g., light levels, increasing cell density) during analysis to detect changes in O_2_^−^ on immediate timescales (i.e., seconds). It has been used to measure O_2_^−^ production in pure cultures (Kustka et al., 2005; Diaz et al., 2013; Schneider et al., 2016; Zhang et al., 2016a; Hansel et al., 2019) and natural waters (Diaz et al., 2016; Hansel et al., 2016; Zhang et al., 2016b). Following the procedures of Diaz et al. (2013), MCLA blanks generated in the absence of SOD were used to determine biologically derived O_2_^−^ concentrations, in order to avoid overestimation of biological O_2_^−^ production rates due to MCLA auto-oxidation. Briefly, cells were deposited onto an inline filter (0.22 μm), continuously rinsed (2 ml min^−1^) with a phosphate buffer (20 mM; pH = 7.6) that matched the salinity of the seawater media base (38 psu), and O_2_^−^ was quantified in the cell-free effluent upon reaction with the MCLA reagent [4 μM MCLA, 0.1 M MES, 75 μM diethylenetriamine pentaacetic acid (DTPA), pH = 6] in a spiral flow cell adjacent to a photomultiplier tube. Chemiluminescent signals from the phosphate buffer and MCLA baselines as well as biological samples were allowed to stabilize (≤4% CV) for at least 1 min, allowing calculation of a steady-state O_2_^−^ concentration. In this way, obtaining a stable chemiluminescent signal from the MCLA reacting with effluent from biological samples demonstrated that the O_2_^−^ being detected was not a rapid, short-lived release of intracellular O_2_^−^ due to cell rupture. SOD was added at the end of each analysis (final concentration of ~800 U l^−1^) to confirm the signal acquired on the FeLume (II) system was due to O_2_^−^. In comparison to the aforementioned study by Diaz et al. (2013), the following exceptions were implemented here. First, cells deposited on the filter were exposed to ambient light (~5 μmol photons m^−2^ s^−1^) unless they were covered in a dark photography film-changing bag to obtain dark (0 μmol photons m^−2^ s^−1^) measurements, as indicated. Second, only net production rates were determined, and finally, calibration was performed using standard additions of potassium superoxide (KO_2_) by the method of Schneider et al. (2016). A preliminary cell concentration was obtained by microscopy using a hemocytometer counting chamber to help ensure that the same number of cells was loaded onto the in-line filter from each biological replicate and on each day within an experiment. The biotic steady-state O_2_^−^ concentration was calculated by subtracting blank signals generated from the mixture of MCLA and phosphate buffer with a syringe filter inline and in the absence of SOD. Then, net O_2_^−^ production rates were calculated by multiplying the biotic steady-state O_2_^−^ concentration (pM) by the flow rate (2 ml min^−1^), dividing that value by the number of cells loaded onto the inline filter (either found using microscopy or flow cytometry), and converting to final units of amol cell^−1^ h^−1^. All chemicals used to measure O_2_^−^ were obtained from Millipore Sigma, except for MCLA, which was obtained from Tokyo Chemical Industry Co., Ltd.

### Statistical Analyses

All statistical analyses were performed using JMP Pro 13.0.0 (SAS Institute Inc., Cary, NC). Regression analyses of cell-normalized O_2_^−^ production rates as a function of time across the growth curve of *E. huxleyi* and cell density were performed using Spearman’s rank-order correlation. This regression analysis indicates the presence or absence of monotonic relationships based on the correlation coefficient (*ρ*) and its level of significance (*p*). An independent two sample Student’s *t*-test was used to determine potential differences between mean *F_v_/F_m_* values measured on various days throughout the growth curve of *E. huxleyi*. To determine the effect of SOD additions on *in vivo* fluorescence, a mixed factor repeated measures ANOVA was used. To determine the effect of dilution on per-cell O_2_^−^ production and the effect of SOD addition on growth rates, cell abundances, cell biovolume, and *F_v_/F_m_* values, a comparison of means using an independent two sample Student’s *t*-test was employed for each parameter interrogated. A one-sample Student’s *t*-test was used to determine the potential difference between *E. huxleyi* O_2_^−^ production in the presence and absence of light. For all statistical analyses, the significance threshold (alpha) was set to 0.05.

## Results

### Extracellular O_2_^−^ Production as a Function of Growth Phase

**Figure 1:**
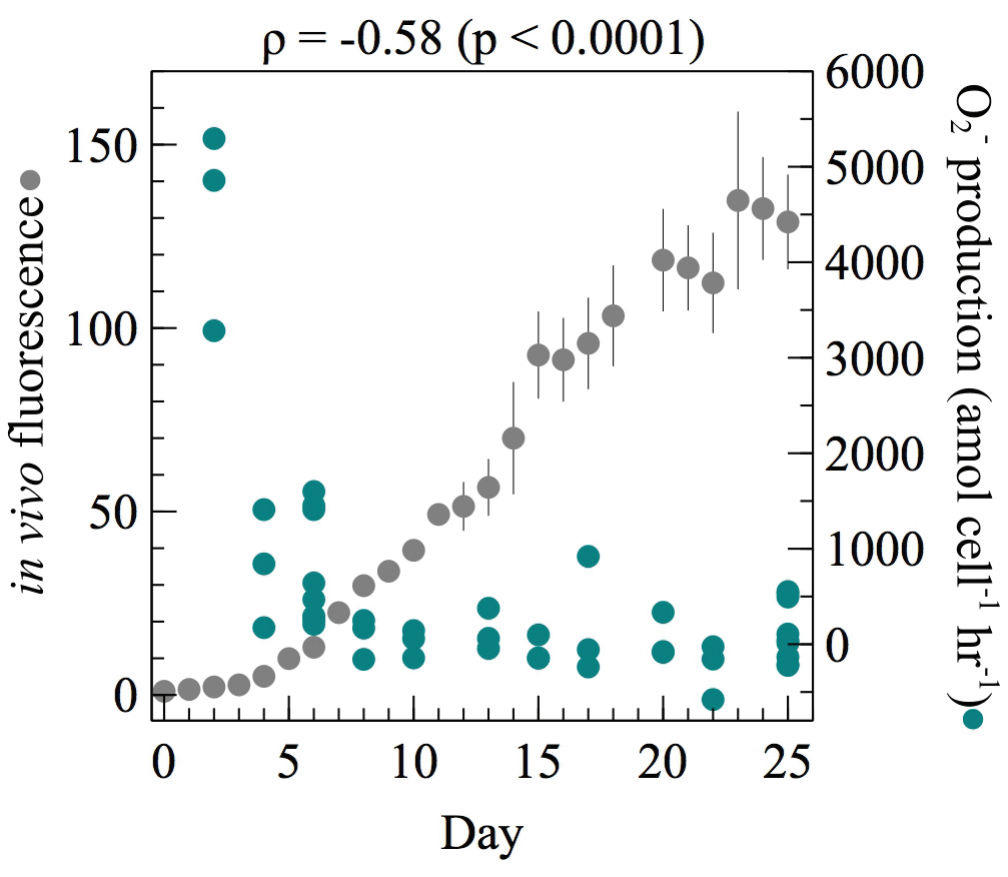
At each time point throughout the growth curve of *E. huxleyi*, net per-cell O_2_^−^ production rates were measured from ~10^6^ cells (*n* = 3 biological replicates for each day except day 6 and day 25 where three separate measurements were made on three biological replicates giving *n* = 9). Regression analysis on per-cell O_2_^−^ production as a function of time was performed using Spearman’s rank-order correlation. The correlation coefficient (*ρ*) and its level of significance (*p*) are provided. *In vivo* fluorescence was normalized to the value on day 0. Error bars indicate one standard error of the mean of three biological replicates.

To assess per-cell extracellular O_2_^−^ modulation across different average metabolic states, cell-normalized extracellular O_2_^−^ production by *E. huxleyi* was measured throughout the growth curve of batch cultures. To rule out potential cell density effects, the number of cells analyzed at each time point was kept constant [average ± SE was 8.32 × 10^5^ ± 5.98 × 10^4^ cells (*n* = 30)]. The highest per-cell net extracellular O_2_^−^ production rates were observed during early exponential growth when *F_v_/F_m_* values were the highest (Supplementary Figure S1) and significantly declined as *E. huxleyi* grew over time (Spearman’s *ρ* = −0.58; *p* < 0.0001) (Figure 1). For example, *E. huxleyi* produced maximum amounts of O_2_^−^ (average ± SE) at the first time point measured in early exponential phase (4,478 ± 611 amol cell^−1^ h^−1^, *n* = 3; day 2). These maximal rates were nearly 78 times higher than net production rates measured between day 8 and day 25, when average production declined to 58 ± 55 amol cell^−1^ h^−1^ (*n* = 30). Flow cytometry analyses revealed this decline was not due to an increase in senescent cells (i.e., cells with low chlorophyll). After day 8, net cell-normalized O_2_^−^ production rates were occasionally negative, fluctuating between −253 ± 167 (day 22; *n* = 3) and 209 ± 360 (day 17; *n* = 3). Net per-cell O_2_^−^ production rates account for the simultaneous production and decay of O_2_^−^ at the cell surface. Because auto-oxidation of the MCLA probe results in a small amount of O_2_^−^ production (Fujimori et al., 1993), the negative net per-cell O_2_^−^ production rates between day 8 and day 25 reflect degradation of O_2_^−^ originating from the MCLA reagent.

### Extracellular O_2_^−^ Production as a Function of Cell Density

**Figure 2:**
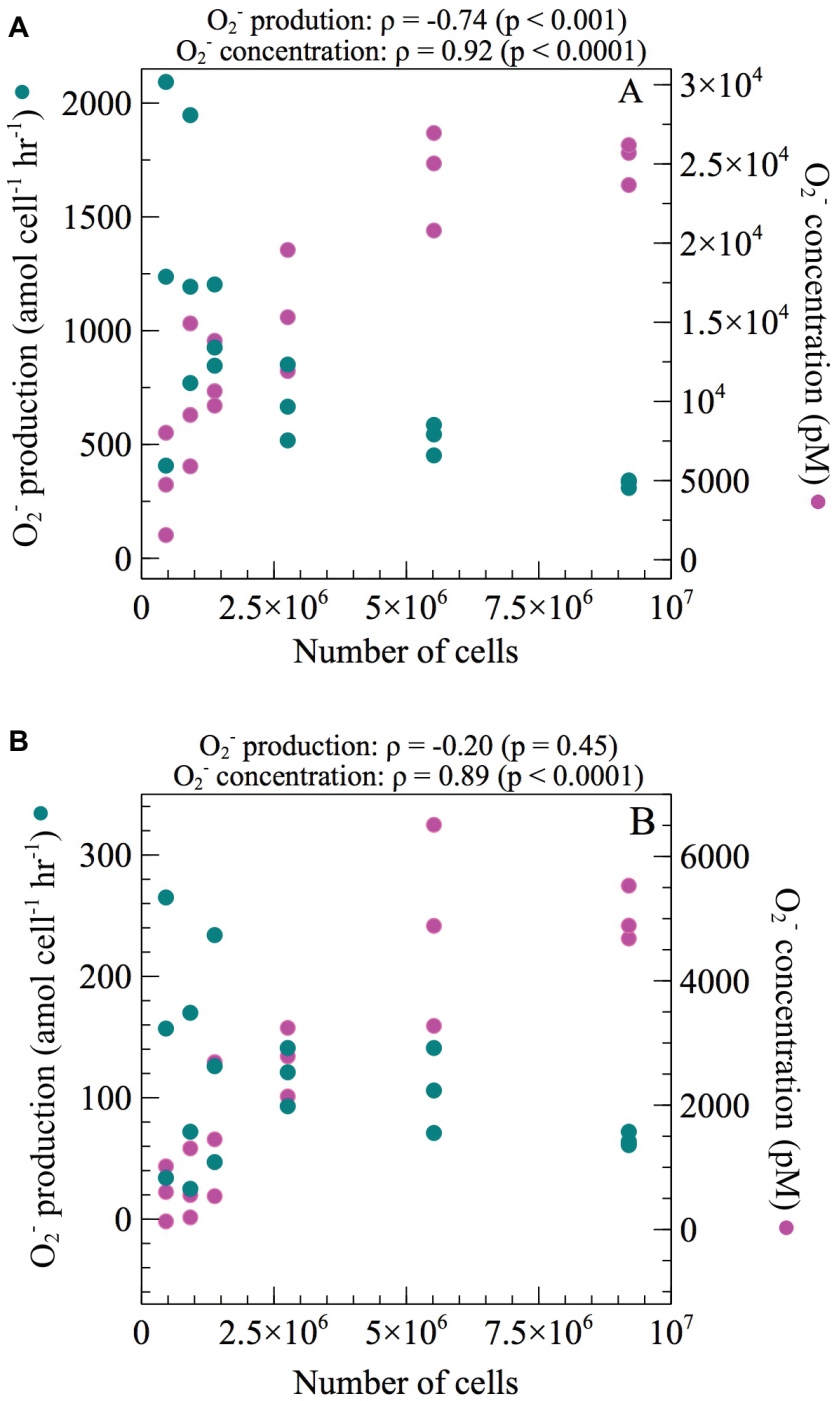
Net per-cell O_2_^−^ production rates and total steady-state O_2_^−^ concentrations were measured across a range of increasing cell numbers during **(A)** exponential and **(B)** stationary growth phases of *E. huxleyi*. Regression analysis was performed using Spearman’s rank-order correlation. Correlation coefficients (*ρ*) and their level of significance (*p*) are provided.

Two approaches were undertaken to determine the potential effect of cell density on extracellular O_2_^−^ production. First, short-term effects (sec-min) were tested by measuring cell-normalized O_2_^−^ production rates and total O_2_^−^ concentrations while increasing the number of cells loaded on the FeLume filter in both exponential and stationary growth phase. The total O_2_^−^ concentration increased significantly with increasing cell density during both exponential (Spearman’s *ρ* = 0.92; *p* < 0.0001) and stationary phase (Spearman’s *ρ* = 0.89; *p* < 0.0001). Conversely, net per-cell O_2_^−^ production rates decreased significantly with increasing cell density during exponential phase (Spearman’s *ρ* = −0.74; *p* < 0.001) but not during stationary phase (Spearman’s *ρ* = −0.20; *p* = 0.45) (Figure 2). In exponential phase, average net per-cell O_2_^−^ production rates decreased by more than 200% from the highest (9.2 × 10^6^) to lowest (4.6 × 10^5^) number of cells analyzed.

**Figure 3:**
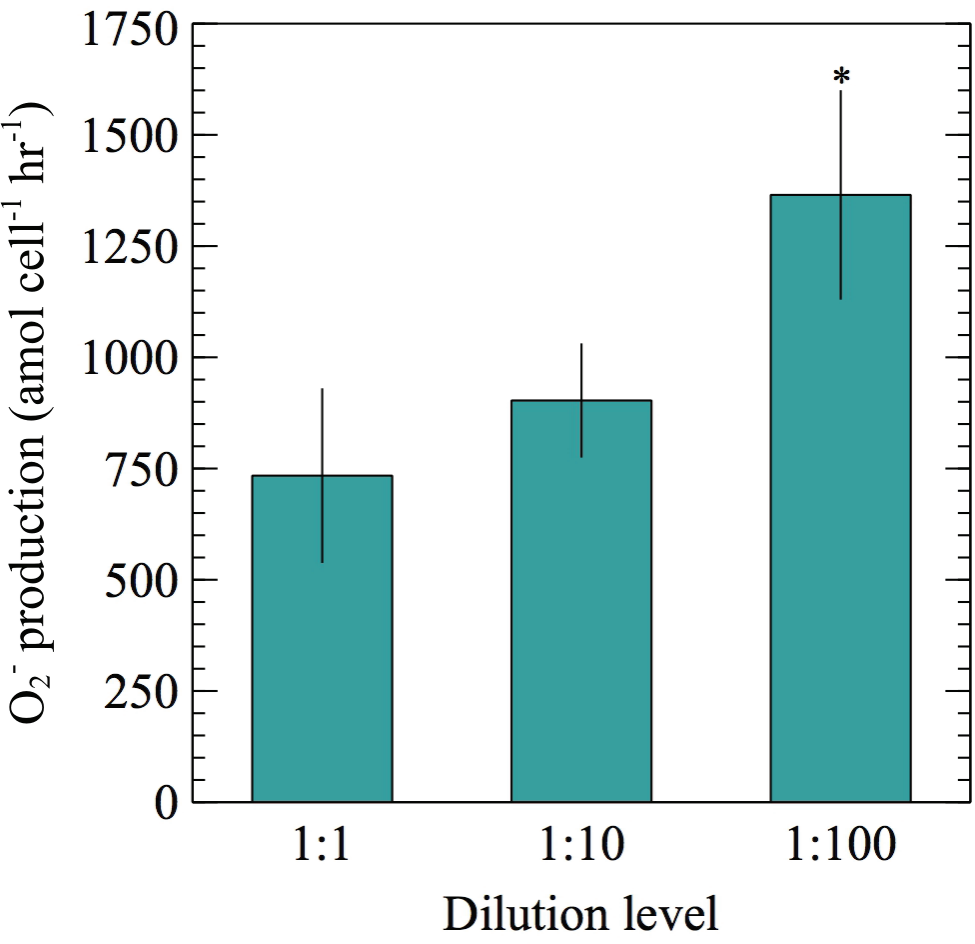
Net per-cell O_2_^−^ production rates were measured from ~10^6^ *E. huxleyi* cells during exponential growth phase post no dilution (1:1), 10-fold dilution (1:10), or 100-fold dilution (1:100) with 0.22 μm filtered, autoclaved seawater for 0–6.5 h. Significant differences (two sample Student’s *t*-test) relative to the undiluted control (1:1) are indicated by asterisks, where *p* < 0.05 is represented by * symbol. Error bars depict one standard error of the mean of replicates (*n* = 8 for 1:100 and 9 for 1:1 and 1:10 dilutions).

To assess longer-term effects (min-h) of cell density on extracellular O_2_^−^ production, exponentially growing cells were preconditioned to lower cell densities by diluting cultures with 0.22 μm filtered, autoclaved seawater and incubating for 0–6.5 h prior to conducting O_2_^−^ measurements. In this experiment, the number of cells loaded on the FeLume filter at each dilution level was kept constant [average ± SE was 1.32 × 10^6^ ± 8.47 × 10^4^ cells (*n* = 26)]. A 10- and 100-fold dilution of *E. huxleyi* resulted in a 51 and 172% increase in cell-normalized net extracellular O_2_^−^ production rates, respectively. Although average rates increased at both dilution levels, only the 100-fold dilution resulted with significantly more extracellular O_2_^−^ per cell than the undiluted control (*t*-test; *p* < 0.05) (Figure 3). The 0.22 μm filtered, autoclaved seawater diluent was also measured for O_2_^−^ to ensure the O_2_^−^ measured in the diluted samples was not due to the seawater diluent. At maximum, O_2_^−^ produced in the seawater diluent could only account for 3.0–4.6% of the total steady-state O_2_^−^ concentrations measured in experiments with *E. huxleyi*, confirming that the contribution from the diluent was negligible.

### SOD Addition Experiments

**Figure 4:**
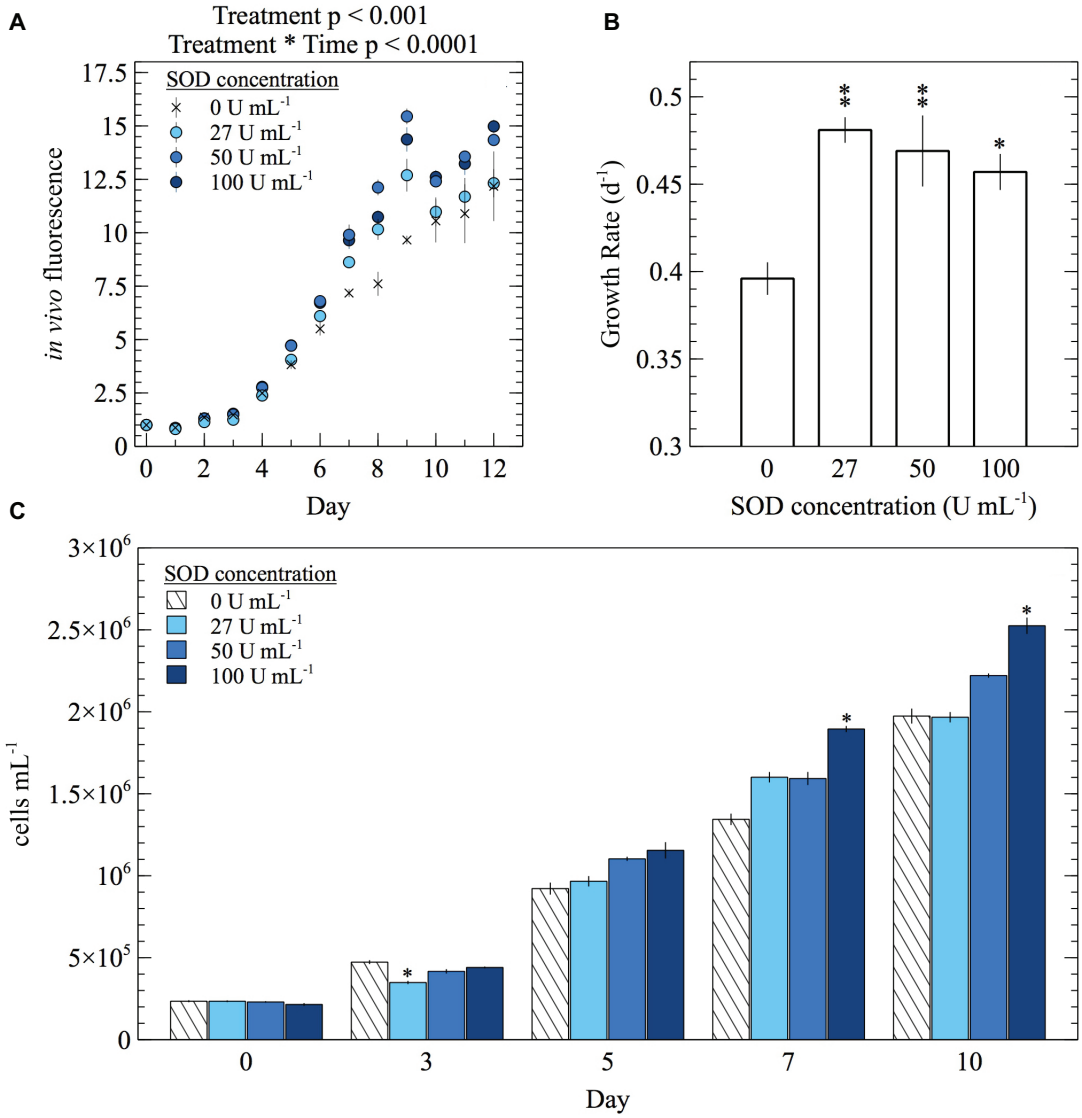
The effect of daily SOD additions on **(A)** average *in vivo* fluorescence, **(B)** specific growth rate during exponential growth phase, and **(C)** cell abundance of *E. huxleyi*. Significant differences in *in vivo* fluorescence between SOD additions were found using a mixed factor repeated measures ANOVA. Significant differences (two sample Student’s *t*-test) in specific growth rate and cell abundances relative to the control (0 U ml^−1^ SOD) are indicated by asterisks, where *p* < 0.05 and < 0.01 are represented by * and ** symbols, respectively. Error bars depict one standard error of the mean of biological replicates (*n* = 3).

To assess how extracellular O_2_^−^ influences growth and physiology, *E. huxleyi* cultures were grown with a range of SOD concentrations and monitored until the end of exponential phase. Since SOD is a large enzyme (>31 kDa) (Cass, 1985), it cannot passively cross cell membranes and therefore selectively targets O_2_^−^ within the extracellular milieu. Overall, the addition of SOD stimulated growth (Figure 4; Supplementary Figure S2; Supplementary Table S2). For example, *in vivo* fluorescence was significantly different in cultures with various SOD concentrations (mixed factor repeated measures ANOVA; *p* < 0.001) and became more significant over time (mixed factor repeated measures ANOVA; *p* < 0.0001) (Figure 4A; Supplementary Table S2). In addition, specific growth rates were between 15 and 22% higher (*t*-test; *p* < 0.05) in the presence of SOD (Figure 4B; Supplementary Table S2). Cell abundances from cultures grown with 100 U ml^−1^ SOD were significantly higher (*t*-test; *p* < 0.05) than cell abundances from cultures grown without SOD beginning on day 7 (Figure 4C; Supplementary Table S2), when cell concentrations in the highest SOD addition were 41% higher than the unamended treatment.

To confirm whether these results were specifically due to SOD, a control experiment was performed in which SOD was removed via diafiltration and only the low molecular weight fraction (<10 kDa) of the SOD suspension was added to cultures. *In vivo* fluorescence was significantly different between treatments (mixed factor repeated measures ANOVA; *p* < 0.05) with significance increasing over time (mixed factor repeated measures ANOVA; *p* < 0.0001) (Supplementary Figure S2A; Supplementary Table S2). Cultures grown with SOD grew significantly faster (*t*-test; *p* < 0.05) than those grown with dialyzed SOD (Figure 2B; Supplementary Table S2). By the end of the control experiment (day 12), cell abundances from cultures grown with dialyzed SOD were significantly lower (*t*-test; *p* < 0.05) than those grown with SOD by about 32% (Supplementary Figure S2C; Supplementary Table S2). Thus, the effect of SOD addition on growth rates and growth yields could not be accounted for by the dialyzed SOD control (Supplementary Figure S2; Supplementary Table S2).

**Figure 5:**
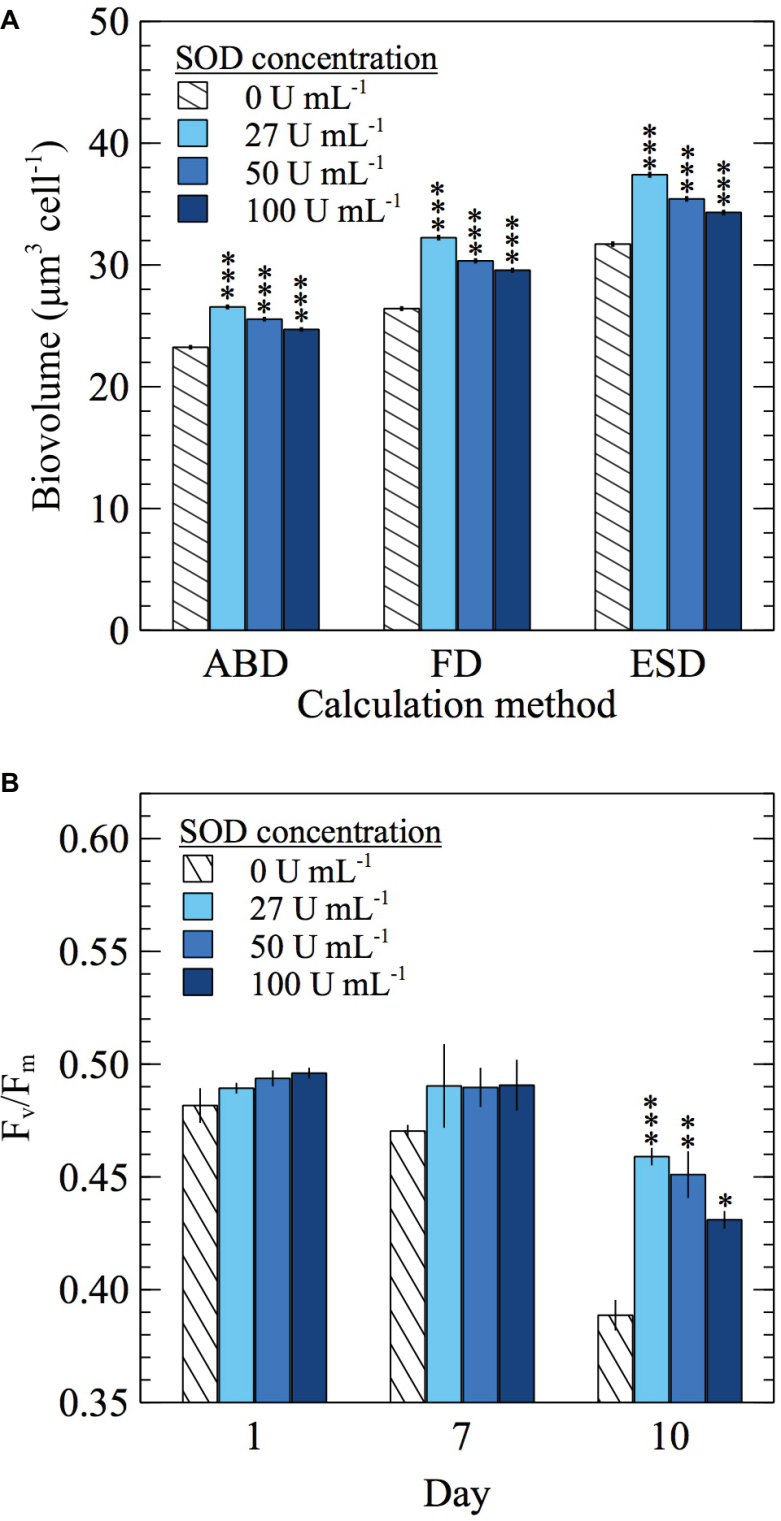
The effect of daily SOD additions on *E. huxleyi* **(A)** cellular biovolume (*n* = 19,041, 17,344, 24,902, and 33,006 individual cells for 0, 27, 50, and 100 U ml^−1^ SOD, respectively) sampled on day 10 and calculated using three different measurements of cell diameter [area based diameter (ABD), filled or full diameter (FD), and equivalent spherical diameter (ESD)] and **(B)** *F_v_*/*F_m_* values (*n* = 3 biological replicates). Significant differences (two sample Student’s *t*-test) relative to the control (0 U ml^−1^ SOD) are indicated by asterisks, where *p* < 0.05, < 0.01, and < 0.0001 are represented by *, **, and *** asterisk symbols, respectively. Error bars represent one standard error of the mean.

In addition to growth yields and growth rates, the cellular biovolume of *E. huxleyi* was monitored in SOD addition experiments. Biovolume was calculated using three different methods, but regardless of the calculation method used, cellular biovolume was larger in cultures grown with SOD (*t*-test; *p* < 0.0001) (Figure 5A; Supplementary Figure S3A; Supplementary Table S2). Adding SOD increased biovolume between 6.3 and 22.1%, depending on the SOD concentration and calculation method used. In the control experiment with dialyzed SOD, biovolume of cells grown with SOD was significantly larger (*t*-test; *p* < 0.0001) than those grown with dialyzed SOD by at least 19% (Supplementary Figure S3A; Supplementary Table S2) confirming the response was due to the presence of SOD.

In addition to stimulating growth, SOD improved photophysiological health, as evidenced by increased *F_v_/F_m_* values, which indicated more efficient light assimilation by PSII in the presence of SOD. By day 10, adding SOD at each concentration increased *F_v_/F_m_* between 11 and 18% compared to the 0 U ml^−1^ SOD control (*t*-test; *p* < 0.05) (Figure 5B; Supplementary Table S2). A similar trend was seen in the control experiment with dialyzed SOD, where *F_v_/F_m_* values from cultures grown with SOD were significantly higher than those from cultures grown with dialyzed SOD starting on day 7 (*t*-test; *p* < 0.05) (Supplementary Figure S3B; Supplementary Table S2). Therefore, improvements in photophysiological health are attributed to the effects of SOD.

### Extracellular O_2_^−^ Production in the Presence and Absence of Light

**Figure 6:**
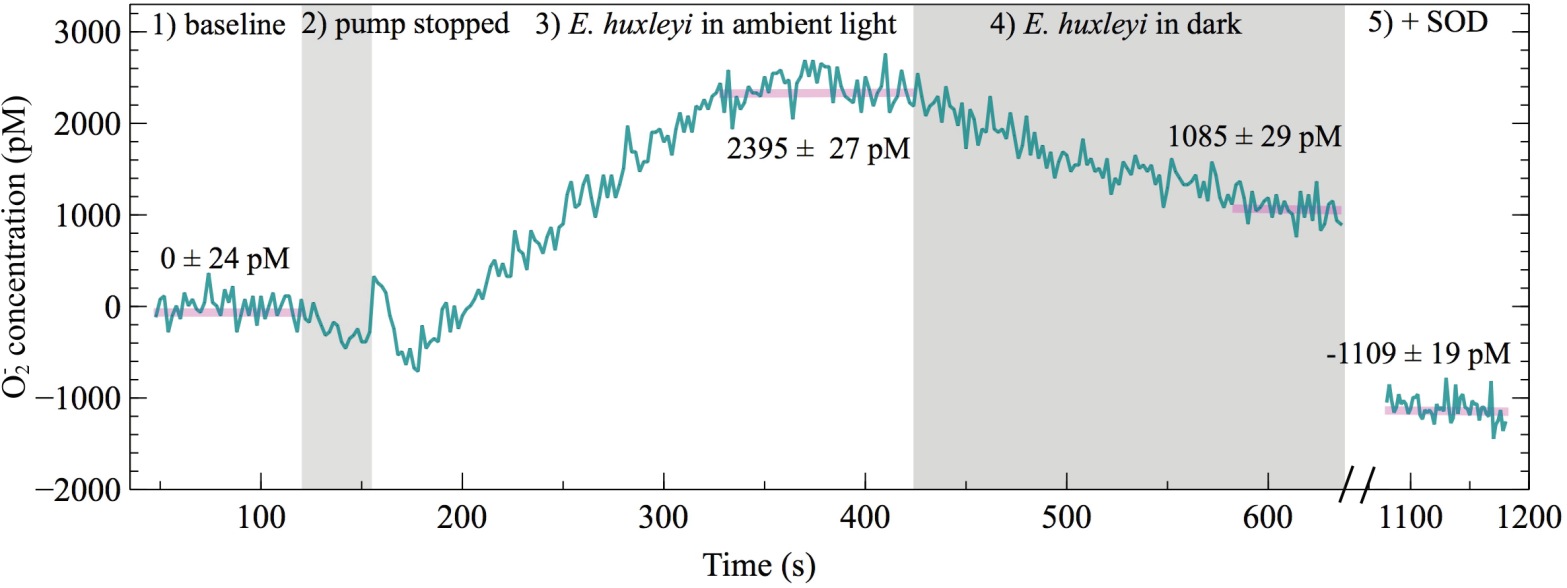
FeLume time-series of O_2_^−^ measurements under different light conditions on day 2 of *E. huxleyi* growth (biological replicate B) split into five regions: (1) phosphate buffer solution and MCLA reagent baseline (which is subtracted from the biogenic O_2_^−^ concentration in regions 3 and 4), (2) shaded region showing loading of *E. huxleyi* cells while the pump was stopped, (3) *E. huxleyi* in ambient light, (4) the second shaded region showing *E. huxleyi* in the dark, and (5) drawdown of the O_2_^−^ signal below the baseline after addition of SOD (negative O_2_^−^ concentrations account for SOD driven degradation of O_2_^−^ originating from auto-oxidation of the MCLA reagent). The average ± SE of stable steady-state O_2_^−^ concentration measurements are indicated by horizontal pink lines.

**Figure 7:**
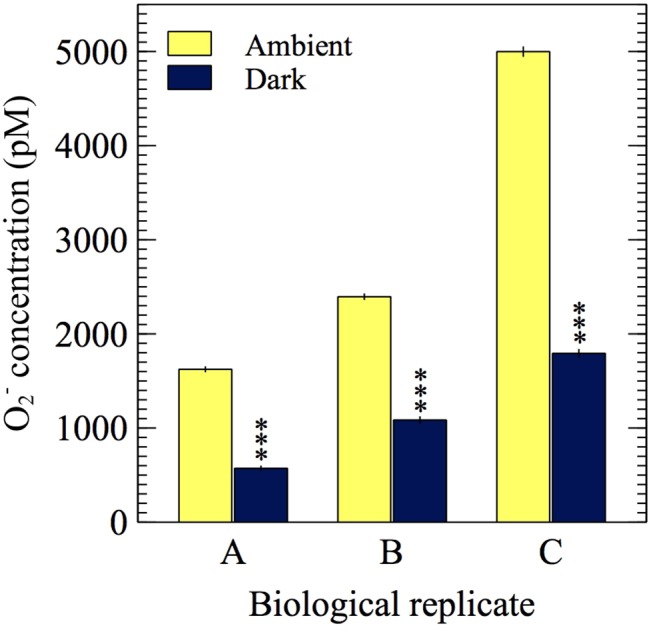
O_2_^−^ measurements from three biological replicates of *E. huxleyi* in the presence of ambient light (~5 μmol photons m^−2^ s^−1^) and in the dark (0 μmol photons m^−2^ s^−1^). Asterisks show significant difference (*p* < 0.0001) between the average dark and average ambient light steady-state O_2_^−^ concentration for each biological replicate. Error bars represent one standard error of the mean (*n* ≥ 31 chemiluminescent counts).

To investigate whether extracellular O_2_^−^ production by *E. huxleyi* is dependent on light, extracellular O_2_^−^ production was measured in ambient light and dark conditions. A representative FeLume time series measurement of O_2_^−^ concentration showed that extracellular O_2_^−^ production by *E. huxleyi* under ambient light reached and stabilized at 2,395 ± 27 pM (Figure 6). Upon removal of light, there was an immediate decline in production, which stabilized at 1,085 ± 29 pM after ~160 s in dark conditions. This result could not be accounted for by abiotic factors, as the removal of light had insignificant effects on O_2_^−^ production in the absence of *E. huxleyi* cells. The drawdown of signal below the abiotic O_2_^−^ baseline by SOD confirmed that the biogenic signal was indeed due to O_2_^−^ production (Figure 6). All biological replicates produced less O_2_^−^ in dark compared to ambient light conditions (*t*-test; *p* < 0.0001), indicating a connection with photophysiological processes (Figure 7). Dark conditions inhibited O_2_^−^ production by an average of 70%.

## Discussion

Here, we investigated the cellular regulation and potential physiological roles of extracellular O_2_^−^ production in a noncalcifying strain (CCMP 374) of the cosmopolitan marine coccolithophore *E. huxleyi* to approach a broader understanding of the potential ecological and environmental impacts of phytoplankton-derived extracellular O_2_^−^. The ability to produce extracellular O_2_^−^ is widespread among phytoplankton, yet production rates can vary substantially within and between species (Diaz and Plummer, 2018). For example, compared to *Chattonella* spp., the most prolific microbial ROS producers, maximal extracellular O_2_^−^ production rates by *E. huxleyi* were about 100–10,000 times lower. Indeed, *E. huxleyi* produced O_2_^−^ at a rate more similar to nonharmful algae such as *Symbiodinium* spp. and *Thalassiosira* spp. (Diaz and Plummer, 2018).

The extracellular O_2_^−^ concentrations and production rates by *E. huxleyi* were not due to the release of intracellular O_2_^−^. First, the release of intracellular O_2_^−^ through cell lysis would have been detected as a rapidly decaying pulse of O_2_^−^, but this can be ruled out because O_2_^−^ signals were stable for at least 1–2 min (see section “Materials and Methods”), a significant timeframe compared to the typical half-life of O_2_^−^ in our analysis (~2 min) (Diaz et al., 2013). Second, the physiochemical nature of the O_2_^−^ anion prevents it from passively crossing intact cell membranes (Bielski et al., 1985; Lesser, 2006; Brown and Griendling, 2009). Therefore, the production rates measured in this study reflect active production of O_2_^−^ on or near the surface of *E. huxleyi*. The reported rates of extracellular O_2_^−^ production reflect the balance of gross production and decay at the cell surface, thus giving a net production rate. Therefore, any change in the net production rate of extracellular O_2_^−^ could result from a change in gross production, decay, or both. Several abiotic and biotic factors have the potential to degrade extracellular O_2_^−^ at or near the cell surface, such as interactions with trace metals including soluble and mineral-bound iron (Fujii et al., 2006; Heller and Croot, 2010a) and expression of cell surface SODs (Aguirre et al., 2005; Oshikawa et al., 2010; Bauer, 2014).

ROS production is commonly associated with stress; however, *E. huxleyi* produced extracellular O_2_^−^ without any added stressors. In fact, cell-normalized O_2_^−^ production by *E. huxleyi* was the highest in early exponential phase under presumably the least taxing culture conditions when nutrient concentrations were the highest (Figure 1) and when photosynthetic efficiency was at a maximum (Supplementary Figure S1). These results reflect the rates of extracellular O_2_^−^ production by cells that were removed from the prevailing culture conditions and analyzed *ex situ*. The *ex situ* analytical conditions such as pH (7.6) were identical across culture samples of all ages, yet differed from *in situ* levels (Supplementary Figure S4). These results therefore show that as cultures age, there is a shift toward lower O_2_^−^ production when cells are analyzed under the same conditions. The potential effect of pH on extracellular O_2_^−^ production by *E. huxleyi* is unknown, but an increase in pH stimulates extracellular ROS production by *C. marina* (Liu et al., 2007), which is opposite to the trend reported here (Figure 1; Supplementary Figure S4). The observed decline in extracellular O_2_^−^ production with culture age therefore suggests that extracellular O_2_^−^ production is physiologically driven and unlikely related to a stress response, as levels of stress would presumably increase with time in batch culture due to the depletion of resources. These findings do not rule out the potential for *E. huxleyi* to upregulate extracellular O_2_^−^ under stressful conditions, as seen with extracellular H_2_O_2_ (Evans et al., 2006) and intracellular ROS (Evans et al., 2006; Vardi et al., 2012) during viral infection, but do demonstrate a physiological investment in the production of extracellular O_2_^−^ under ideal growth conditions, which suggests some role in basal metabolism. In order to clarify if and how *E. huxleyi* regulates extracellular O_2_^−^ in response to biogeochemical variability and stress, future work should consider the influence of factors such as viral infection and pH.

In addition to having growth phase dependence (Figure 1) similar to other phytoplankton (Oda et al., 1995; Kim et al., 1999; Portune et al., 2010), cell-normalized net extracellular O_2_^−^ production rates by *E. huxleyi* were also inversely dependent on cell density over a range of timescales during exponential growth phase. For example, this trend occurred when cell density increased on time scales of seconds to minutes (Figure 2A) and when cells were preconditioned to lower cell densities on timescales of minutes to hours (Figure 3). This tight regulation may suggest a dynamic cell density-dependent signaling role for O_2_^−^ production in *E. huxleyi*. For instance, extracellular O_2_^−^ deriving from one cell may act as a signal between other cells or within the same cell to provide information on surrounding population density (Diaz and Plummer, 2018). Similar cell density dependent trends in extracellular O_2_^−^ production have been demonstrated in other phytoplankton (Marshall et al., 2005a; Hansel et al., 2016; Diaz et al., 2018), including *C. marina* (Marshall et al., 2005b).

Although evidence has been accumulating that extracellular O_2_^−^ production is directly involved in growth promotion in a range of microbial cell types (Oda et al., 1995; Aguirre et al., 2005), our results show that the presence of extracellular O_2_^−^ does not directly stimulate growth of *E. huxleyi*. The potential role of extracellular O_2_^−^ in *E. huxleyi* growth was addressed through selective removal of extracellular O_2_^−^ from the local environment of cells using SOD. Scavenging O_2_^−^ promoted growth (Figure 4), increased cell biovolume (Figure 5A), and improved photosynthetic efficiency (Figure 5B). Conversely, removal of extracellular O_2_^−^ from cultures of *Chattonella* spp. attenuates growth (Tanaka et al., 1992; Oda et al., 1995). Specifically, in one prior study, *C. marina* growth was significantly hampered under similar SOD concentrations used in the present study, and the morphological state of cells was altered (Oda et al., 1995). Recently, Hansel et al. (2019) revealed that the growth of common marine bacteria from the *Roseobacter* clade was inhibited by SOD in a dose-dependent manner (Hansel et al., 2019). Similarly, the removal of extracellular ROS from fungi and the amoebozoan *Dictyostelium discoideum* is also detrimental to development (Aguirre et al., 2005). Taken together, extracellular O_2_^−^ does not seem to directly stimulate growth in *E. huxleyi*. Rather, these results may highlight a different role for extracellular O_2_^−^ in *E. huxleyi* that contrasts with the proposed growth-promoting role of extracellular O_2_^−^ in *C. marina* (Oda et al., 1995), bacteria (Hansel et al., 2019), fungi, and protists (Aguirre et al., 2005). However, the addition of SOD not only removes O_2_^−^ but produces H_2_O_2_, which may also have impacts on *E. huxleyi* physiology. For example, high concentrations of H_2_O_2_ are harmful to phytoplankton (Dupouy et al., 1985; Morris et al., 2011), but normal growth of *C. marina* is dependent on low levels of extracellular H_2_O_2_ (Oda et al., 1995). In fact, in a variety of cell types, the dismutation of Nox-derived O_2_^−^ by cell surface SOD generates extracellular H_2_O_2_, which can diffuse into the cell, to elicit gene expression (Shapiguzov et al., 2012), morphogenesis (Rossi et al., 2017), and proliferation (Oshikawa et al., 2010; Bauer, 2014). It remains possible, yet speculative, that the addition of SOD in our experiments accelerated the dismutation of O_2_^−^ to H_2_O_2_, which then may have acted as a growth promoter for *E. huxleyi*. Thus, the role of extracellular O_2_^−^ in *E. huxleyi* may be contingent on its ability to give rise to H_2_O_2_, which should be interrogated in future work.

The fact that there are clear trends in extracellular O_2_^−^ production as a function of growth phase and cell density, but that *E. huxleyi* growth was not stunted with removal of extracellular O_2_^−^, leaves the possibility open that extracellular O_2_^−^ could be connected to other aspects of *E. huxleyi* physiology and health. To examine whether extracellular O_2_^−^ production may be involved in photosynthetic physiology, we interrogated O_2_^−^ production as a function of light and found that O_2_^−^ production was attenuated within seconds upon transition from light to dark conditions (Figure 6). This finding adds to a growing body of evidence linking modulation of extracellular O_2_^−^ production by phytoplankton to light availability and therefore photophysiology. For instance, extracellular O_2_^−^ production is light dependent in many phytoplankton including *Thalassiosira* spp. (Milne et al., 2009; Schneider et al., 2016), *Trichodesmium* (Hansel et al., 2016), *Symbiodinium* (modulated on the same time scales shown here) (Saragosti et al., 2010), and *Chattonella* spp. (Kim et al., 1999; Marshall et al., 2002; Dorantes-Aranda et al., 2013). Thus, O_2_^−^ production may somehow be involved with light dependent processes (e.g., photosynthesis, photoacclimation physiology), and this functionality may be conserved across phytoplankton taxa. Interestingly, in a previous study, when *C. marina* was treated with DCMU [3-(3,4-dichlorophenyl)-1,1-dimethylurea], an electron transfer inhibitor between photosystem II and I, extracellular O_2_^−^ production was quenched to levels observed under dark conditions (Marshall et al., 2002), further illustrating a mechanistic link between extracellular O_2_^−^ production and photophysiology.

Overall, this study reveals that the stress-independent production of extracellular O_2_^−^ by *E. huxleyi* is dynamically regulated, and potentially part of a basal process involved with photophysiology. Extracellular O_2_^−^ production by *E. huxleyi* is conceivably part of healthy cellular functioning for several reasons. First, the fact that cells diverted energy toward making O_2_^−^ in the absence of a stressor suggests its production can be unassociated with stress and probably related to basal functioning. Indeed, *E. huxleyi* produced maximum amounts of extracellular O_2_^−^ per cell under ideal growth conditions while cells were most metabolically active (Figure 1) and when efficiency of photosynthetic processes was the highest (Supplementary Figure S1). Further, the steady-state concentrations of O_2_^−^ generated by *E. huxleyi* cells are not consistent with concentrations that would be damaging (>10^−6^ M) but are consistent with concentrations of biological signaling molecules (~10^−12^ M) (Saran, 2003). Additionally, *E. huxleyi* cells tightly controlled the production of extracellular O_2_^−^ as a function of light, cell density, and growth phase, and on timescales as short as seconds, consistent with other phytoplankton (Oda et al., 1995; Kim et al., 1999; Marshall et al., 2002, 2005a,b; Milne et al., 2009; Portune et al., 2010; Saragosti et al., 2010; Dorantes-Aranda et al., 2013; Hansel et al., 2016; Schneider et al., 2016; Diaz et al., 2018), suggesting a potential dynamic role in signaling and photophysiology. Indeed, stress-independent extracellular O_2_^−^ production by *E. huxleyi* is in agreement with several other microorganisms such as phytoplankton and bacteria where its production is prolific under ideal growth conditions (Oda et al., 1995; Kustka et al., 2005; Rose et al., 2005; Marshall et al., 2005a,b; Godrant et al., 2009; Portune et al., 2010; Diaz et al., 2013; Hansel et al., 2016; Schneider et al., 2016). The fact that *E. huxleyi* still produces extracellular O_2_^−^ in the absence of light suggests there could be additional and/or alternative purposes for this production beyond photophysiology. Indeed, extracellular O_2_^−^ production can be produced through a variety of subcellular mechanisms and could be produced for a combination of ecophysiological functions (Diaz and Plummer, 2018). Contrasting with other diverse microorganisms (Tanaka et al., 1992; Oda et al., 1995; Saran, 2003; Aguirre et al., 2005; Hansel et al., 2019), the presence of extracellular O_2_^−^ does not promote growth in *E. huxleyi*. This finding underscores a potentially unprecedented role for the presence and/or production of extracellular O_2_^−^, which should be investigated among other phytoplankton. Finally, non-calcifying *E. huxleyi* cells coexist with calcareous varieties in nature but typically are not dominant (Frada et al., 2012). Several ecological and physiological processes are variable within (Strom et al., 2003; Strom and Bright, 2009; Sunda and Hardison, 2010; Harvey et al., 2015; Poulson-Ellestad et al., 2016) and between (Paasche, 2002; Suggett et al., 2007; Harvey et al., 2015; Poulson-Ellestad et al., 2016) calcifying and noncalcifying strains. Whether the rates, regulation, and roles of extracellular O_2_^−^ production are different in calcifying versus noncalcifying strains of *E. huxleyi* has yet to be determined but should be considered in future work.

## Data Availability

Any materials and data will be made available to members of the scientific community upon request.

## Author Contributions

SP and JD conceived the study. SP conducted the experiments and data analysis, with contributions from AT. All authors contributed to interpretation of results and preparing the manuscript.

## Conflict Of Interest Statement

The authors declare that the research was conducted in the absence of any commercial or financial relationships that could be construed as a potential conflict of interest.
