# Supplementary material for: Dynamic Regulation of Extracellular Superoxide Production by the Coccolithophore Emiliania huxleyi (CCMP 374)
Source: Front Microbiol. 2019 Jul 12;10:1546. doi: 10.3389/fmicb.2019.01546 (PMC6640029; doi:10.3389/fmicb.2019.01546)
Supplement: Supplementary file 1 [file Data_Sheet_1.pdf]

Supplementary Material

1 Supplementary Methods

1.1 Analysis and filtering of FlowCam® images

To filter out all non-*E. huxleyi* particles, two universal sort functions were applied to every sample. First, each sample was sorted for filled pixel area. The area of the smallest and largest *E. huxleyi* cell in each sample was noted. Then, each sample was sorted for Hu Circularity. Hu Circularity assigns a value from 0 to 1 for how well the edge trace of the particle fits a perfect circle (1). Since *E. huxleyi* is spherical and appears circular in two dimensions, this was deemed an effective method for removing pieces of contaminants such as transparent exopolymer particles (TEP). The value of the lowest and highest *E. huxleyi* Hu Circularity in each sample was noted. Then, each sample was filtered for these two parameters (i.e., filled pixel area, Hu Circularity). Particles that did not fit the parameters were binned and not included in further analysis. The sample was then scanned visually for extraneous particles and any found were removed.

Biovolume was calculated with 3 different measurements of cell diameter: Area Based Diameter (ABD), Filled or Full Diameter (FD), and Equivalent Spherical Diameter (ESD). ABD is found by measuring the number of pixels within a circular-traced cell, determining the area of the cell from the measured number of pixels, and finding diameter from this calculated area. ESD is calculated by taking the average value of 36 feret measurements, where a feret measurement is the distance between two parallel lines touching either side of the cell. FD is the diameter determined from a cell that has an area equal to the maximum filled intracellular area.

Reference

1. Žunić J, Hirota K, Rosin PL. 2010. A Hu moment invariant as a shape circularity measure. Pattern Recognition 43:47-57.

Supplementary Table 1. FlowCam® context parameters for *E. huxleyi* imaging

FlowCam® Context Parameter	Setting
Objective	20x Olympus
Collimator	10x/20x Collimator Installed
Sample Volume	500 µL
Flow Rate	0.02 mL min^{-1}
Dilution	0.2
Distance Between Cells	0 µm
Filter	Use equivalent spherical diameter (ESD), from 2 µm to 20 µm.
Laser	Enable
Channel 1 and 2	ON, threshold 400, measure ON
Scatter	ON, measure
Fluorescence Scale	Linear
Sample Width	4
Flash Duration	100 µsec
Flash Delay	1 µsec

Supplementary Table 2. The effect of various concentrations of SOD (Experiment Number 1), as well as SOD, DI, and dialyzed SOD (Experiment Number 2) on parameters assessing growth and physiology of *E. huxleyi*. Day number indicates the sampling day for each parameter assessed. Biovolume was calculated with 3 different measurements of cell diameter: Area Based Diameter (ABD), Filled or Full Diameter (FD), and Equivalent Spherical Diameter (ESD). Averages and standard errors of the mean of 3 (except for biovolume) biological replicates are given.

Experiment Number	Treatment	Concentration (U mL^{-1})	Specific Growth Rate (d^{-1})	Cell Abundance		Biovolume			F_v/F_m	
				Day	(cells mL^{-1})	Diameter	(µm^3 cell^{-1})	n	Day	—
**1**	**SOD**	0	0.396 ± 0.009	0	$2.35 \times 10^5 \pm 2.88 \times 10^3$	ABD	23.242 ± 0.055	19402	1	0.482 ± 0.007
				3	$4.73 \times 10^5 \pm 8.65 \times 10^3$	FD	26.416 ± 0.064		7	0.470 ± 0.002
				5	$9.22 \times 10^5 \pm 4.65 \times 10^4$	ESD	31.713 ± 0.088		10	0.389 ± 0.006
				7	$1.34 \times 10^6 \pm 3.16 \times 10^4$					
				10	$1.97 \times 10^6 \pm 4.23 \times 10^4$					
	**SOD**	27	0.481 ± 0.013	0	$2.34 \times 10^5 \pm 2.13 \times 10^3$	ABD	26.554 ± 0.064	17345	1	0.489 ± 0.002
				3	$3.49 \times 10^5 \pm 6.45 \times 10^3$	FD	32.241 ± 0.094		7	0.490 ± 0.18
				5	$9.66 \times 10^5 \pm 2.83 \times 10^4$	ESD	37.409 ± 0.107		10	0.459 ± 0.004
				7	$1.60 \times 10^6 \pm 2.85 \times 10^4$					
				10	$1.97 \times 10^6 \pm 2.83 \times 10^4$					
	**SOD**	50	0.469 ± 0.018	0	$2.30 \times 10^5 \pm 1.65 \times 10^3$	ABD	25.548 ± 0.053	24903	1	0.494 ± 0.003
				3	$4.17 \times 10^5 \pm 1.02 \times 10^3$	FD	30.337 ± 0.075		7	0.490 ± 0.008
				5	$1.10 \times 10^5 \pm 9.66 \times 10^3$	ESD	35.423 ± 0.092		10	0.451 ± 0.10
				7	$1.59 \times 10^6 \pm 3.68 \times 10^4$					
				10	$2.22 \times 10^6 \pm 9.66 \times 10^3$					
	**SOD**	100	0.457 ± 0.010	0	$2.15 \times 10^5 \pm 5.22 \times 10^3$	ABD	24.708 ± 0.047	33007	1	0.496 ± 0.002
				3	$4.41 \times 10^5 \pm 2.43 \times 10^3$	FD	29.569 ± 0.065		7	0.491 ± 0.011

Experiment Number	Treatment	Concentration (U mL^{-1})	Specific Growth Rate (d^{-1})	Cell Abundance		Biovolume			F_v/F_m	
				Day	(cells mL^{-1})	Diameter	(μm^3 cell^{-1})	n	Day	—
				5	$1.16 \times 10^5 \pm 4.65 \times 10^4$	ESD	34.314 ± 0.081		10	0.431 ± 0.007
				7	$1.90 \times 10^6 \pm 1.53 \times 10^4$					
				10	$2.53 \times 10^6 \pm 4.65 \times 10^4$					
**2**	**DI**	—	0.406 ± 0.014	0	$1.72 \times 10^5 \pm 7.48 \times 10^3$	ABD	27.333 ± 0.056	49007	1	0.486 ± 0.002
				3	$3.39 \times 10^5 \pm 5.02 \times 10^4$	FD	37.610 ± 0.087		4	0.543 ± 0.002
				5	$8.38 \times 10^5 \pm 4.84 \times 10^4$	ESD	42.946 ± 0.098		7	0.509 ± 0.003
				7	$1.79 \times 10^6 \pm 1.43 \times 10^5$				10	0.495 ± 0.000
				10	$2.58 \times 10^6 \pm 2.12 \times 10^5$				12	0.482 ± 0.006
				12	$3.21 \times 10^6 \pm 1.24 \times 10^5$					
	**Dialyzed SOD**	—	0.416 ± 0.006	0	$1.77 \times 10^5 \pm 1.46 \times 10^3$	ABD	26.664 ± 0.047	61757	1	0.493 ± 0.002
				3	$4.60 \times 10^5 \pm 3.50 \times 10^4$	FD	37.305 ± 0.075		4	0.535 ± 0.002
				5	$1.13 \times 10^6 \pm 4.47 \times 10^4$	ESD	42.482 ± 0.083		7	0.517 ± 0.003
				7	$2.14 \times 10^6 \pm 1.13 \times 10^4$				10	0.508 ± 0.001
				10	$3.12 \times 10^6 \pm 9.80. \times 10^4$				12	0.494 ± 0.003
				12	$3.35 \times 10^6 \pm 1.99 \times 10^5$					
	**SOD**	100	0.439 ± 0.004	0	$1.74 \times 10^5 \pm 4.17 \times 10^3$	ABD	31.603 ± 0.045	96758	1	0.488 ± 0.001
				3	$4.03 \times 10^5 \pm 2.27 \times 10^4$	FD	46.606 ± 0.072		4	0.540 ± 0.006
				5	$1.07 \times 10^5 \pm 2.90 \times 10^4$	ESD	53.052 ± 0.081		7	0.541 ± 0.003
				7	$2.29 \times 10^6 \pm 1.60 \times 10^5$				10	0.517 ± 0.005
				10	$4.08 \times 10^6 \pm 2.26 \times 10^5$				12	0.528 ± 0.009
				12	$4.41 \times 10^6 \pm 1.33 \times 10^5$					

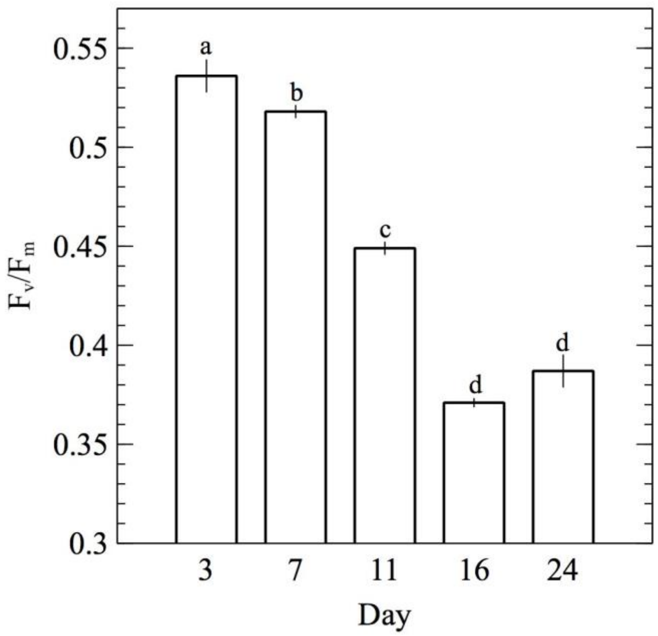

Supplementary Figure 1. F_v/F_m values measured at various points along the growth curve of *E. huxleyi*. Significant differences (comparison of the means, two sample student's t-test) between average F_v/F_m values on each day are indicated by different letters. Error bars represent one standard error of the mean of biological replicates (n = 3).

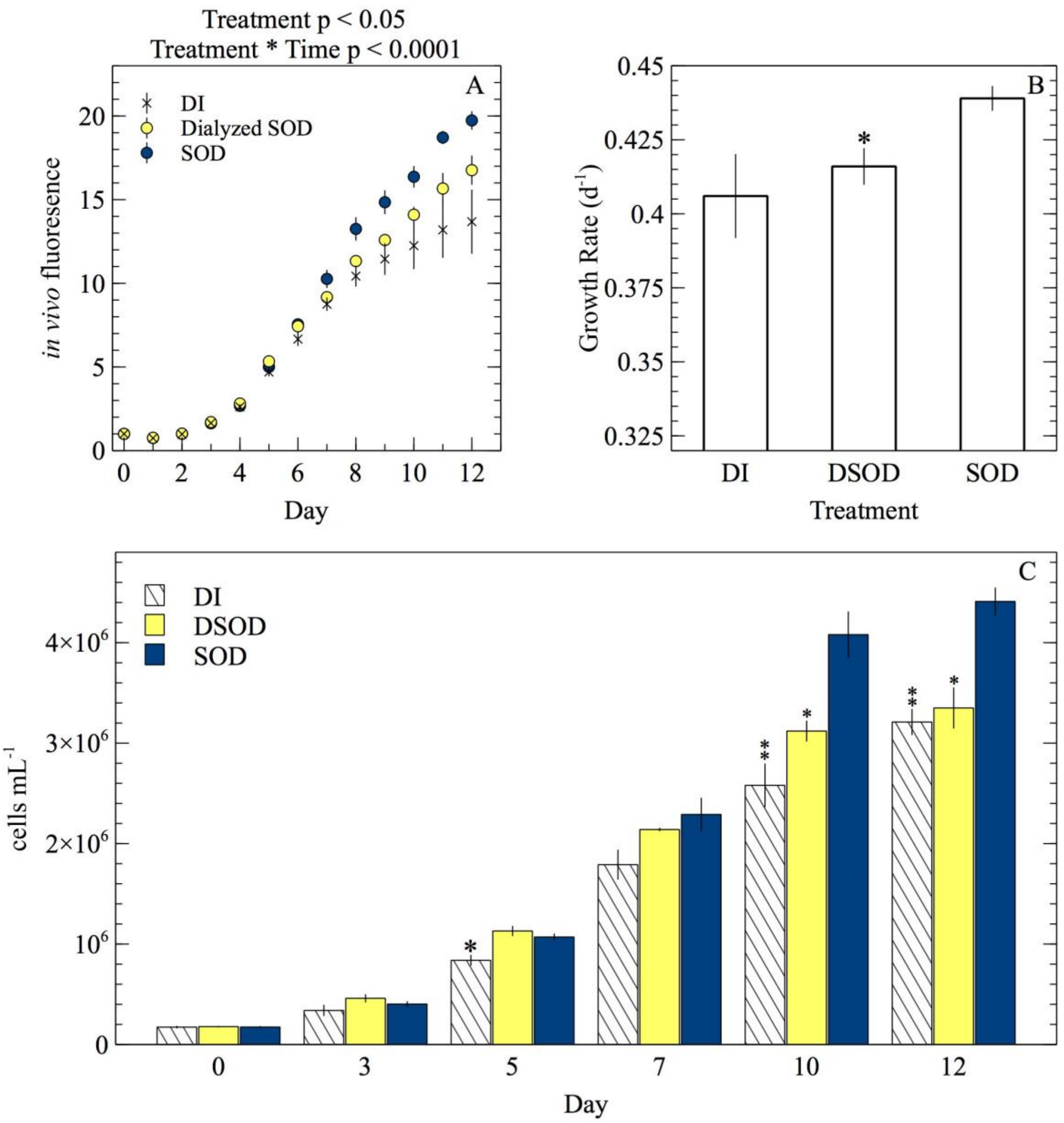

Supplementary Figure 2. The effect of daily additions of DI, dialyzed SOD (DSOD), and 100 U mL^{-1} SOD on (A) average *in vivo* fluorescence, (B) specific growth rate, and (C) cell abundance of *E. huxleyi*. Significant differences in *in vivo* fluorescence between SOD additions were found using a mixed factor repeated measures ANOVA. Significant differences (two sample student's t-test) in specific growth rate and cell abundances relative to the 100 U mL^{-1} SOD treatment are indicated by asterisks, where a p-value of < 0.05, < 0.01, and < 0.0001 are represented by one, two, and three asterisk symbols, respectively. Error bars depict one standard error of the mean of biological replicates (n = 3).

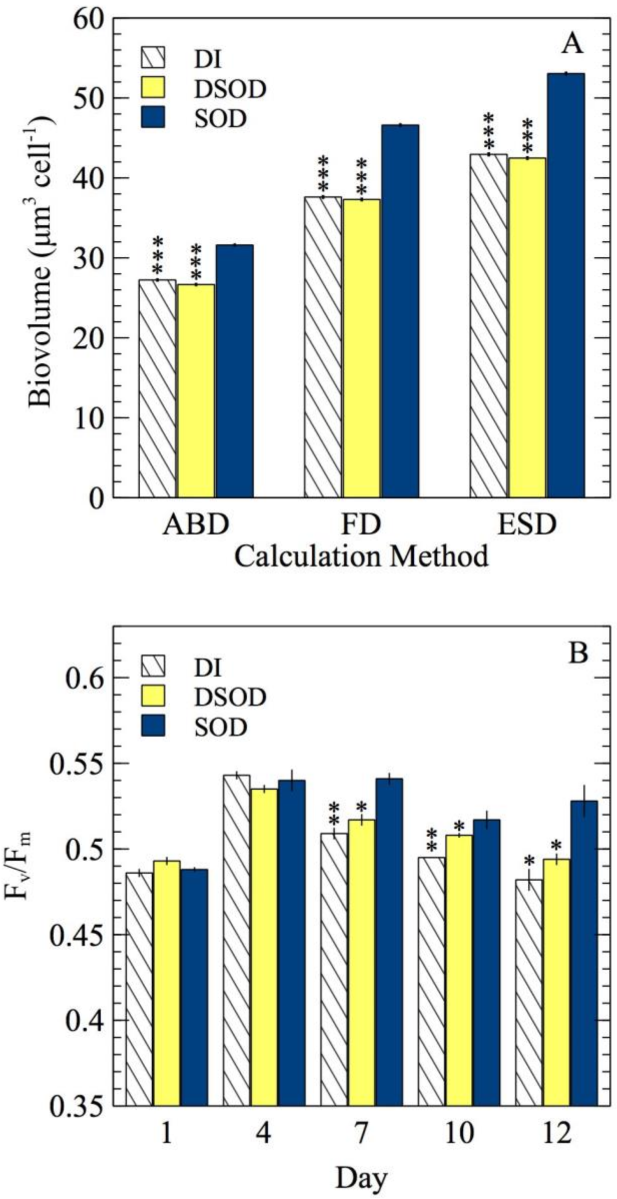

Supplementary Figure 3. The effect of daily additions of dialyzed SOD (DSOD), DI, and 100 U mL^{-1} SOD on *E. huxleyi* (A) average cellular biovolume (µm^3 cell^{-1}; n = 49007, 61757, and 96758 for DI, dialyzed SOD, and 100 U mL^{-1} SOD, respectively) sampled on day 10 and calculated using 3 different measurements of cell diameter (Area Based Diameter (ABD), Filled or Full Diameter (FD), and Equivalent Spherical Diameter (ESD)) and (B) F_v/F_m values (n = 3 biological replicates). Significant differences (two sample student's t-test) relative to the 100 U mL^{-1} SOD are indicated by asterisks, where a p-value of < 0.05, < 0.01, and < 0.0001 are represented by one, two, and three asterisk symbols, respectively. Error bars represent one standard error of the mean.

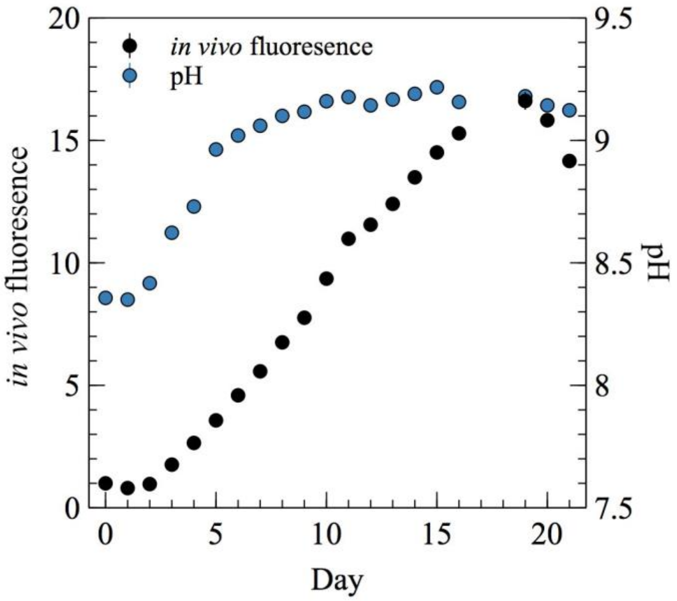

Supplementary Figure 4. The average pH and *in vivo* fluorescence of *E. huxleyi* cultures (n=3) were measured throughout the growth curve. Most of the error bars, representing one standard error of the mean, are hidden by the data symbols.
